# Municipal Hospital Care of Contagious Diseases*Read by invitation before the San Antonio District Medical Society, San Antonio, Texas, October 25, 1910, and contributed to the Texas Medical Journal.

**Published:** 1910-11

**Authors:** A. W. Fly

**Affiliations:** Galveston, Texas


### TEXAS MEDICAL JOURNAL.

Established July, 1885

F. E. DANIEL, M. D., - - - - - - Editor, Publisher and Proprietor

Published Monthly.—Subscription $1.00 a Year.

VOL. XXVI AUSTIN, NOVEMBER, 1910. No. 5

The publisher is not responsible for the views of the contributors.

### Original Articles.

For Texas Medical Journal.

### Municipal Hospital Care of Contagious Diseases.*

BY A. W. FLY, M. D., GALVESTON, TEXAS.

Of all the notable achievements in the realm of science during the Nineteenth century, none have been so pregnant with the welfare and happiness of mankind as those of scientific medicine, especially as regarding the prevention of diseases. In earlier times but little attention was paid to man's most potent enemy, the disease germ, but sickness was considered a necessary evil whose origin was thought to lay in human nature like qualities and inclinations which develop, more or less, according to surroundings and habits of the individual. The doctor paid attention only to a possible healing or easement of the patient, not knowing the cause; there was only a limited chance of cure. Since, however, the cause of these contagious diseases has been discovered, a more and more growing realization has taken place,—that prevention of epidemic conditions is more economical than to take up the fight, after the contagion has taken a foothold. During the past fifty years preventive medicine has done more to alleviate suffering and prolong life than people in general realize. About fifty years ago the general death rate was 80 to 90 per thousand, with an alarming increase during epidemics up to 75 per cent of all infected. If we

*Read by invitation before the San Antonio District Medical Society, San Antonio, Texas, October 25, 1910, and contributed to the Texas Medical Journal.

compare these figures with statistics of the present day, and take the death rate of large cities like London, Berlin and New York, the average is only between 17 and 18, and no one will assert that people have been growing stronger during these strenuous times. This condition is due principally to the preventive measures employed at the present time against infectious diseases, and, at the same time, the proper education in the care of the child during the first five years of his life.

In speaking, therefore, of contagious diseases, in general, and of their care in municipal hospitals, we will especially dwell on the essential means to prevent further infection, beside the medical treatment of the disease itself.

One of the main reasons why only rarely during epidemic cures of the contagious diseases were obtained was, that the only place where the contagious diseases were treated was the home of the infected. It was, therefore, not possible to give the cases proper care, as a trained nurse and a doctor could not watch all the patients at all times. The hospitals, private as well as public, did not take any contagious diseases on account of the danger of contagion to other patients and, therefore, home treatment was the only possible way of caring for them. However, when more doctors realized the importance of preventing the spreading of such diseases, they found the care of such infected patients in public hospitals of the greatest importance to the welfare of all communities. We find that, then, municipalities considered the care of contagious infection as one of their great duties, and this question is, at the present time, a burning one.

In the proper treatment of contagious diseases, it is of the greatest importance that a separate building be provided for the treatment of contagious diseases only. If possible, it should be on a high, sightly point outside of the city; this will afford the patients good pure air not infected by smoke and dust of the city. However, we do not believe that there is the least danger to a neighborhood from a well conducted sanatorium for contagious diseases, at such institutions; owing to the careful hygienic and educational features of the surroundings, it is hardly likely to be contracted at all.

For the care of consumptives, all sleeping and resting quarters face the south, and are fully exposed to air and light, with only screen wire separating them from the outside air.

Quarters for diseases that infect by contact, as smallpox, etc., must be enclosed and arranged for the regulation of air, light and temperature. There must be separate kitchens for the patients, and, better yet, separate kitchens for smallpox and consumptive patients; also, the ward for smallpox, scarlet fever, etc., should be separated absolutely from the consumptive.

So much in regard to the building of our municipal hospitals.

There is no disease of such disastrous effect as consumption (or tuberculosis). The disease, which is preventable and curable, may infest itself in any part of the body,—in the lungs, throat, intestines, meninges or bones and joints. The presence of the germ of tuberculosis, in the living tissue of man or animal, causes a growth of new cells, which unite and form a tubercle; from that the name of tuberculosis is derived. A tubercle may be defined as a minute nodule, or elevation, often not larger than a pinhead, and of rather clear and vesicular appearance. All tuberculosis diseases are locally characterized by countless numbers of such tubercles, which are visible to the naked eye. The tubercle is made up of various kinds of cells; from their resemblance to other ordinary epithelial cells, are often called epitheloid cells. The bacilli usually lodge in the giant cells of the tubercle; they can be found by the millions in the affected organs. It is this little parasite which must be considered as the specific cause of tuberculosis. The micro-organism of tuberculosis, as found in man, is a short, narrow, slightly curved rod with rounded and pointed ends. Tubercle bacilli grow best at a temperature of 98 degrees Fahrenheit, but withstand extreme degrees of cold and a considerable amount of heat. Fresh air and light are inimical to them. The secretions from these different micro-organism are called toxins, and are the cause of serious symptoms, such as fever, night sweats, etc. The disease is most prevalent in its pulmonary form; as such, it has been known for hundreds of years as the most feared, most prevalent and, also, the most fatal of diseases.

Some of the old doctors believed in the contagiousness of the disease, but it was left to the old French physician, Villimin, to demonstrate beyond a doubt that tuberculosis could be transmitted from one individual to another. The discovery of the specific organism (bacillus tuberculosis) was made by the great scientist, Robert Koch, in 1882. According to some statistics, every sixth human being in America and Europe is afflicted with tuberculosis, and every seventh death is due to consumption. It may perhaps safely be said that of all mankind 12 per cent die of tuberculosis of one form or another. Tuberculosis, in America, is most prevalent among the colored race, the mortality being nearly double that of the white population.

The average annual tribute of the United States to this scourge is over 100,000 of its inhabitants. The civilized world, it is estimated, yields up every year over 1,000,000; every day, about 3000; each minute, two of its people, as a sacrifice to this plague. In the matter expectorated by a consumptive in four hours the number of bacilli has been estimated at seven billion. The deadly tuberculi can enter the human, or animal, system in three ways: First, by being inhaled into the lungs; second, by being ingested; that is, eaten with tuberculosis food; third, by inoculation; that is, by the penetration of tuberculous substances through the skin, through a wound or cut. The infection by inhalation takes place in the following manner: The tuberculous expectoration, or spittle, is carelessly deposited on the floor; it dries, becomes pulverized and mingles with the dust of the air and then is inhaled. The inhalation, or ingestion, of the small particles of saliva, which may be expelled when speaking quickly, or loud, or when sneezing, must also be considered dangerous for those who come in close contact with them. These almost invisible drops of saliva contain tubercle bacilli. Recent experiments show clearly the possibility of infection by these means.

From the following we can take what means have to be employed to give proper care to the patients in municipal hospitals:

There must be isolation, as much as possible, to lessen the possibility of infection. The consumptives must be held in rooms which give pure air and light, unrestricted access, as pure air and light are the greatest enemies of the death-bringing bacilli. Let the rooms for these patients be absolutely outside, only shielded by mosquito netting against insects, which not only bring discomfort, but also carry away the germs of infection. Let the patients be in the free air day and night, summer and winter. For comfort, provide them with woolen blankets to protect them from the cold in the winter. Let them further be provided with the best nourishing food, such as eggs, milk, vegetables, beefsteak, and these in the greatest abundance six times a day, for it has been proven that by this method the bacillus can not thrive, but is smothered to death by the rich food. There must be the most exacting cleanliness, as dust and dirt are the breeding places of the germs.

To protect against infection, the greatest care must be taken in regard to expectorations. The patients must be provided with separate vessels, filled partly with water, to prevent the propagation of the disease from the droplets expelled during dry coughing. All that is necessary is to hold the hand, or handkerchief, before one's mouth, and wash the hands before touching food. When the patient is careful in the disposal of his sputum, and during the act of coughing, he is as safe an individual to associate with as a healthy person.

We think it of great importance that there should be an office provided, in which everyone who may think himself infected shall have free access for a thorough examination, because the earlier the disease can be attacked the greater is the hope of a cure; in the early stages the disease can be mastered, but in the latter stages it is absolutely hopeless.

The second method of infection, arising from food, such as tuberculous milk and meat, can be avoided by not using raw milk or meat when not absolutely sure that the source is pure. In all doubtful cases submit the milk to sterilizing, and the meat to thorough cooking. Whenever possible the hospital should have its own dairy, where everything can be watched. When this disease is discovered in its early stage, as many as 75 or 85 per cent can be cured, when only fresh air, rest, sunshine, baths, careful breathing exercises and good food, with the help of certain medical substances, to assist the proper digestive organs, need be the only means employed. It requires climatic changes,—you can have all the above in any locality.

The next important contagious disease to be considered is smallpox. It is a highly infectious and eruptive fever, said to have caused,.during the Eighteenth century, one-tenth of the total mortality. Smallpox has an incubation period of twenty-four hours to twelve days. An attack is ushered in by chills, rise of temperature, headaches, vomiting and violent pains in the loins. Three days later an eruption develops and the temperature falls more or less, but rises again about the eighth day after the first symptoms. The eruptions first show as pimples on the head or at the margin of the hair; next they appear on the hands and legs, and a little later spread over the whole body. Next, the pimples turn to vesicles, full of lymph; in about eight days these become pustular and with suppuration the temperature rises again. About eleven days after the first symptoms crusts begin to form and the temperature to fall again. Convalescence is then usually uninterrupted.

From this disease there is danger of contagion from the time of taking it until all crusts have fallen off. The other attendants may carry the infection to others without having the disease themselves. The general treatment of smallpox is that of all fever; immediate isolation is the first step; there should be a separate building, remote from all the others,—best about 400 feet distant, or, if in the same building, absolutely separated from the others. The best and safest remedy in this disease is vaccination. It is a much proven fact that those vaccinated are practically immune against infection in its most serious forms, as the confluent and the disastrous form of the hemorrhagic which is nearly in all cases fatal. There must be the necessary provisions as to cleanliness here, principally as to the excreta and cloth, and the parties in attendance. Any sign of eye trouble must at once be met, guarding the eyes from light, and by the frequent use of antiseptic lotions, such as boracic lotion. Oily ointments are believed to be helpful during and after pustulation in aiding the removal of the crust, and lessen the distressing irritation. Many methods are advocated for the prevention of disfigurement by pitting, but if the pustules are deep they will leave scars in spite of all care. Finsen recommends that the sufferer be exposed to red light only; others recommend pricking the pustules at an early stage.

The lighter contagious diseases, such as scarlet fever and measles, are easily handled. These cases should only be admitted to a municipal hospital when conditions at home do not give the most essential points to be considered, as pure air, clean surroundings and right diet.

In conclusion, we will speak of one condition we think should be considered most carefully. We mean the doctor as medium of infection himself. A modern doctor will consider it bad policy to visit a consumptive, or a scarlet fever patient, and, without change of apparel, enter a public conveyance and come in contact with all kinds of people, who are more or less apt to be contaminated. We are sorry to say we know of cases where doctors have been treating diphtheria, and even smallpox, and afterward go on their regular practice, thus exposing other patients to contagion.

We consider it the duty of every faithful doctor to wear separate clothing at the home, and in the presence of a patient with a contagious disease from what he wears in his own home. Let doctors themselves become advocates in this way who do not only preach to others, but set a good example themselves, and we will be able, with the united force of medicine and clean habits, to reduce the mortality brought about through contagious diseases to a very small per cent. And with the present rapid concentration of population in the cities, the demand for hospital accommodations will steadily increase, and so, also, will the demand for municipal regulation of such hospitals. This will mean continued improvement in their sanitary aspects, the limitation of the spread of contagious diseases and the skilled supervision of food supplies.

I have thus briefly endeavored to lay before you the general plan of the workings of the municipal hospital. It is yet in an early stage of its growth; its ultimate result will be the penetration of all communities and a concentrated action by all against that common foe, the contagious disease.

